# Reduced Cortical Thickness in Primary Open-Angle Glaucoma and Its Relationship to the Retinal Nerve Fiber Layer Thickness

**DOI:** 10.1371/journal.pone.0073208

**Published:** 2013-09-03

**Authors:** Longhua Yu, Bing Xie, Xuntao Yin, Minglong Liang, Alan C. Evans, Jian Wang, Chao Dai

**Affiliations:** 1 Department of Radiology, Southwest Hospital, Third Military Medical University, Chongqing, China; 2 Department of Radiology, 401st hospital of the People’s Liberation Army, Qingdao, Shandong, China; 3 McConnell Brain Imaging Centre, Montreal Neurological Institute, McGill University, Montreal, Quebec, Canada; 4 Ophthalmology research center, Southwest Eye Hospital/Southwest Hospital, Third Military Medical University, Chongqing, China; Medical University Graz, Austria

## Abstract

**Objectives:**

To examine possible changes in cortical thickness and their relationship to retinal nerve fiber layer (RNFL) thickness in patients with primary open-angle glaucoma (POAG).

**Materials and Methods:**

Thirty-six patients with POAG and 40 matched healthy controls were enrolled in this study. All subjects underwent a comprehensive ophthalmologic examination and a high resolution structural magnetic resonance scan. Cortical thickness analysis was used to assess the changes between patients and controls. Correlations between the thickness of the visual cortex and RNFL thickness were also analyzed. Finally, the relationship between the severity of changes in the visual cortex and RNFL thickness was evaluated by comparing patients with mild and severe groups.

**Results:**

POAG patients showed significant bilateral cortical thinning in the anterior half of the visual cortex around the calcarine sulci (left BA 17 and BA 18, right BA17) and in some smaller regions located in the left middle temporal gyrus (BA37) and fusiform gyrus (BA19). The thickness of the visual cortex correlated positively with RNFL thickness (left, r = 0.44, *p* = 0.01; right, r = 0.38, *p* = 0.03). Significant differences between mild and severe groups were observed with regard to both RNFL thickness and the thickness of bilateral visual cortex (*p* < 0.05).

**Conclusion:**

Our findings indicate that cortical thickness analysis may be sufficiently sensitive to detect cortical alterations in POAG and that the measurement has great potential for clinical application.

## Introduction

Primary open-angle glaucoma (POAG) is a progressive optic neuropathy characterized by irreversible loss of retinal ganglion cells and optic nerve fibers, degeneration of the axons in optic nerve (ON), and visual field loss [1]. It is a leading cause of blindness worldwide, and by 2020, it is predicted that approximately 60 million people will suffer from POAG [2].

Recent studies have shown that glaucoma may be a neurodegenerative disease similar to Alzheimer’s or Parkinson’s disease [3,4]. The fundamental common process in such diseases is the death of a specific population of neurons followed by anterograde or retrograde trans-synaptic spread of degeneration (Wallerian degeneration) from abnormal to normal neurons [3]. Animal experiments and human postmortem studies in glaucoma have suggested widespread degeneration from retinal ganglion cells to the lateral geniculate nucleus (LGN) and to the primary visual cortex (BA 17) [5–8]. Additionally, recent structural magnetic resonance imaging (MRI) studies have revealed decreased gray matter (GM) volume or density in the primary visual cortex (BA 17) and the secondary visual cortex (BA 18, BA 19) in patients with POAG [9–11]. Altogether, these previous findings suggest that the entire visual pathway, i.e., not only retinal ganglion cells but also the visual cortex, may be involved in human POAG.

Progressive retinal ganglion cell loss occurs in most cases of glaucoma and is induced by elevated intraocular pressure [12]. Retinal nerve fiber layer (RNFL) thickness is a sensitive parameter for the early detection of glaucoma damage [13] and can be measured by optical coherence tomography (OCT) [14]. In the clinic, OCT is a high-resolution ophthalmic imaging technology that is considered to provide similar findings to pathological examination. However, for visual cortex, reduced cortical thickness has thus far only been demonstrated in a postmortem study of POAG [6]. Several MRI studies on the structural brain changes that occur in POAG have used voxel-based morphometry (VBM), which provides a probabilistic measure of local GM concentration [15]. These studies have demonstrated decreases or increases in GM volume or density in POAG in a number of different focal brain regions, most consistently in the visual cortex [9–11,16]. However, results from other cortical regions are inconsistent. For example, the GM of the middle temporal gyrus was found to be decreased [10], increased [11], or unchanged [9]. Measurement of cortical thickness provides a more direct index of cortical morphology that is less susceptible to positional variance because the extraction of the cortex adheres to the GM surface despite local variations in position [17,18]. To the authors’ knowledge, no other study has used cortical thickness analysis to assess the human brain changes in POAG. Additionally, it remains unclear whether the thickness of visual cortex is correlated with RNFL thickness in patients with POAG.

Therefore, the present study was designed to evaluate possible changes of cortical thickness in patients with POAG using high-resolution MR data, and the correlation between the thickness of visual cortex and RNFL thickness was also investigated. Finally, we split the patients into mild and severe groups to explore the relationship between POAG stage and cortical changes.

## Materials and Methods

### Ethics Statement

All research procedures were approved by the Human Research Ethics Committees of the Third Military Medical University and were conducted in accordance with the Declaration of Helsinki. All participants in our study gave written informed consent.

### Subjects

Thirty-six patients with bilateral POAG were enrolled in this study (Table 1). All patients fulfilled the diagnostic criteria for POAG established by the American Academy of Ophthalmology, which include intraocular pressure ≥ 21 mmHg, a glaucomatous optic disc and visual field abnormalities, and a nonoccludable anterior chamber angle without characteristics of congenital or secondary glaucoma [19]. Exclusion criteria were any other ocular, neurological, or psychiatric disorder; any history or clinical signs of auto-immune disease, cardiovascular disease, cerebrovascular disease, and diabetes mellitus; and hypertension or antihypertensive medication.

**Table 1:** Baseline patient demographics.

**Characteristics**	**Values**
Age (year)	46.5 (18-72)
Male/female	27/9
Best-corrected acuity (decimal)	0.96 (0.6-1.5)
Intraocular pressure	
Highest recorded (mmHg)	28 (17-49)
Cup-to-disc ratio	
Right eye	0.77 (0.21-0.97)
Left eye	0.74 (0.36-0.98)
Visual field MD (dB)	
Right eye	-8.9 (-0.88 to -32.68)
Left eye	-7.0 (-0.33 to -31.76)
RNFL thickness (μm)	
Right eye	63.7 (39-107)
Left eye	67.5 (42-102)
Cumulative bilateral eyes	137.5 (82-206)

Data are shown as the median (range); RNFL, retinal nerve fiber layer; MD, mean deviation.

The control group comprised 40 age-, gender-, and education- matched healthy subjects (22-62 years old; mean age, 46.5 years; 11 women). All controls underwent a comprehensive ophthalmologic examination to exclude glaucoma and other eye diseases. Individuals in both groups were right-handed as assessed using the Edinburgh inventory [20].

### RNFL Thickness and Visual Field Examination

RNFL thickness was measured quantitatively using Topcon 3D OCT-1000 (Topcon Corporation, Tokyo, Japan). The mean RNFL thickness was extracted using the fast RNFL thickness protocol at a distance of 3.4 mm from the center of the optic disc for each of the 4 peripupillary quadrants (inferior, superior, nasal, and temporal). The universal RNFL thickness (mean RNFL thickness score for all quadrants) was also recorded.

Visual fields were recorded using 30-2 program of the Humphrey Visual Field Analyzer (Carl Zeiss Meditec, Dublin, Calif). Qualified tests had a false positive of < 10% and fixation loss and false negative of < 15%. Based on the Hodapp-Anderson-Parrish system [21], patients in stage 0 (mean deviation scores, MD > 0 dB) or stage 5 (unable to perform Humphrey visual fields in the “worst eye”) were not enrolled in this study.

### Mild and Severe Groups

Because of the relatively small sample size (36 patients), a median split was performed to divide the POAG group into two subgroups (a mild and a severe group) to evaluate potential effects on the severity of changes in cortical thickness. The mild group was defined as MD ≥ -12 dB in the poorer performing eye in each patient (stage 1 and stage 2; 18 patients, 18-72 years old; mean age, 42.5 years; 4 women), and the severe group was defined MD < -12 dB in the poorer performing eye in each patient (stage 3 and stage 4; 18 patients, 23-65 years old; mean age, 46.4 years; 5 women).

### MRI Data Acquisition

MR scans were performed on a 3-Tesla scanner (Magnetom Trio, Siemens, Erlangen, Germany) using an 8-channel phased-array head coil. The subjects were required to close their eyes and to avoid any movement during image acquisition. 3D structural data were obtained using a T1-weighted magnetization prepared rapid acquisition gradient echo (MPRAGE) sequence. The parameters were: sagittal continuous no-interval scan covering the whole brain; repetition time, 1,830 ms; echo time, 4.43 ms; inversion time, 1,100 ms; flip angle, 9^°^; matrix, 256 × 256 mm^2^; slices, 176; and voxel size, 1.0 × 1.0 × 1.0 mm^3^.

### MRI Data Analysis

The MR images were processed using the CIVET MRI analysis pipeline (version 1.1.9) developed at the Montreal Neurological Institute (MNI) to automatically extract and co-register cortical surfaces for each subject. The main pipeline processing steps included the steps outlined below(1). The native 3D structural MR image of each subject was corrected for non-uniformity using the N3 algorithm [22] and linearly registered into a MNI152 standard space [23]. (2) Each brain volume was classified as gray matter, WM, cerebrospinal fluid, and background using the INSECT algorithm [24]. (3) The Constrained Laplacian-based Anatomic Segmentation with Proximity (CLASP) algorithm was applied to generate a model of the cortical surface comprising 40,962 vertices for each hemisphere [17]. (4) To obtain accurate cross-subject correspondence, the extracted hemispheric cortical surfaces were nonlinearly aligned to a hemisphere-unbiased iterative surface template [25]. (5) The aligned cortical surfaces were rescaled back to the native-space dimension using the inverse of the scaling parameters of the corresponding linear volumetric transformation matrix. (6) The cortical thickness was measured using the t-link metric by computing the Euclidean distance between linked vertices on the inner and outer cortical surfaces, respectively [26].

### Statistical Analyses

Group comparisons between POAG patients and normal controls were conducted to test for differences in cortical thickness using the Surfstat toolbox (http://www.math.mcgill.ca/keith/surfstat) based on Random Field Theory [27]. Statistical analyses were performed according to the general linear model using age, gender, education level, and brain volume were used as covariates. Clusters were reported reaching a loose significance level of *p* < 0.005, uncorrected. Then the clusters with significant group differences were used as masks, and the mean cortical thickness of each cluster for each participant was calculated by averaging over all vertices within the same mask.

Next, partial correlation analyses between the RNFL thickness and the mean cortical thickness of the significant clusters in POAG patients were conducted while controlling for age, gender, education level, and brain volume. Neural fibers of the nasal half of the retina decussate and join the uncrossed temporal fibers of the opposite nerve to form the visual cortex. Thus, the cumulative RNFL thickness of both eyes was calculated for the correlation analyses [28]. The level of significance for all correlation analyses was set at *p* < 0.05.

Finally, independent-sample t tests were used to evaluate differences in the mean cortical thickness of the clusters between mild and severe groups. Additionally, we compared the bilateral RNFL thickness between mild and severe groups.

## Results

### Differences in Cortical Thickness between Groups

**Figure 1:**
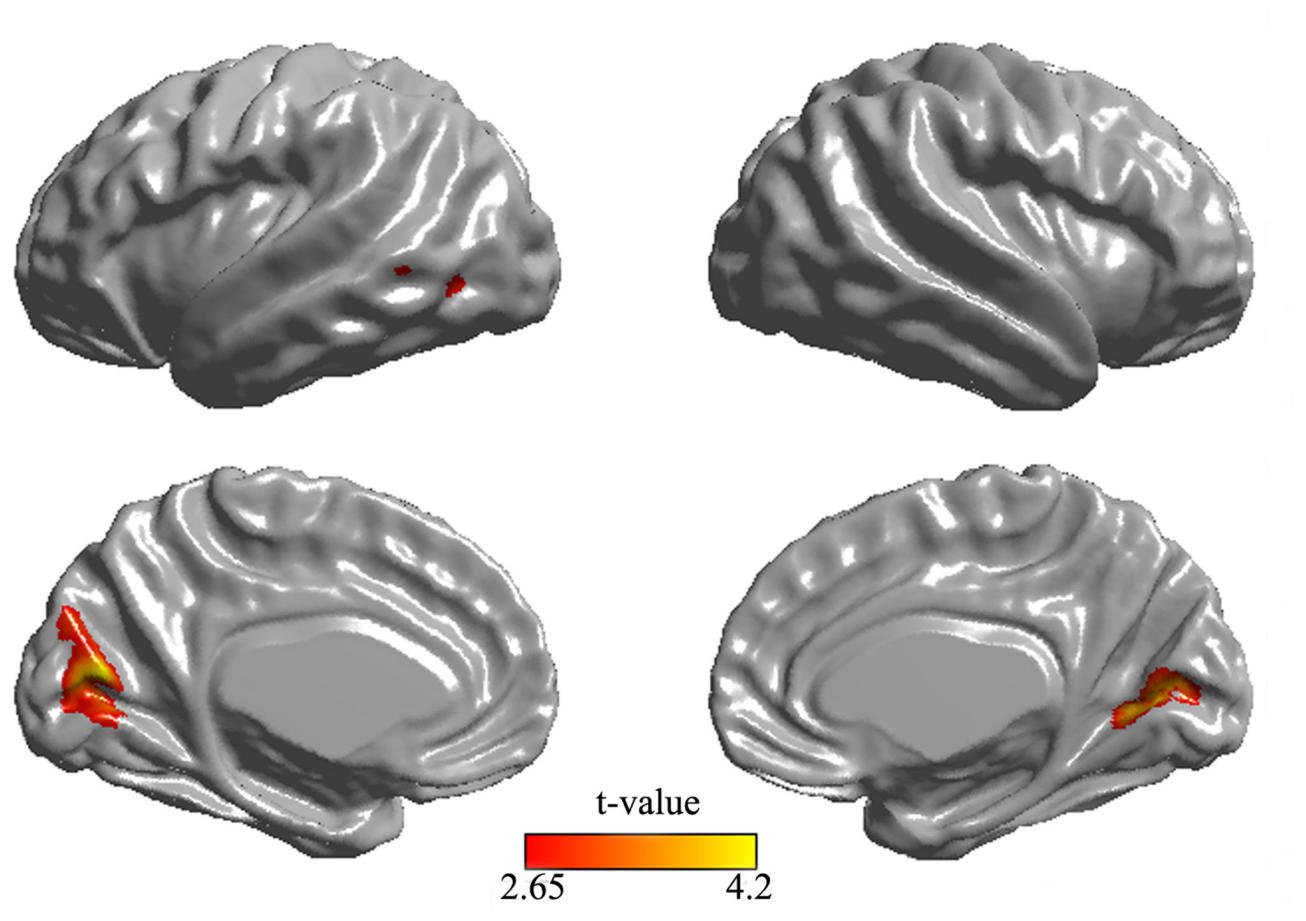
Cortical thinning in POAG patients compare to normal controls. Differences in cortical thickness are superimposed on a hemisphere-unbiased iterative surface template. Color represents the regions of cortical atrophy in patients with POAG.

POAG patients showed significant bilateral cortical thinning in the anterior half of the visual cortex around the calcarine sulci (calcarine cortex) including the right BA 17 and left BA 17 and BA 18. Some smaller regions located in the left middle temporal gyrus (BA37) and the fusiform gyrus (BA19) also showed thinning relative to normal controls (*p* < 0.005, uncorrected; Figure 1, Table 2). No increase in cortical thickness relative to the controls was found in patients.

**Table 2:** Location and size of cortical areas showing significant reductions in thickness in patients with glaucoma relative to controls.

**Regions**	**BA**	**Mean thickness (mm)**		**MNI coordinates**			**Vertex number**	**Peak t-value**
		**NC**	**Patients**	**x**	**y**	**z**		
Left calcarine, cuneus	17, 18	2.64 ± 0.13	2.52 ± 0.15	-3	-68	13	520	4.19
Right calcarine	17	2.75 ± 0.16	2.61 ± 0.16	15	-69	10	365	4.11
Left middle temporal gyrus	37	3.20 ± 0.26	3.03 ± 0.22	-45	-62	-4	39	2.94
Left fusiform gyrus	19	3.22 ± 0.25	3.07 ± 0.24	-35	-75	-12	20	2.85

BA, Brodmann area; NC, normal controls; *p* < 0.005, uncorrected; cluster size > 10.

### Correlations between Cortical Thickness and RNFL Thickness

**Figure 2:**
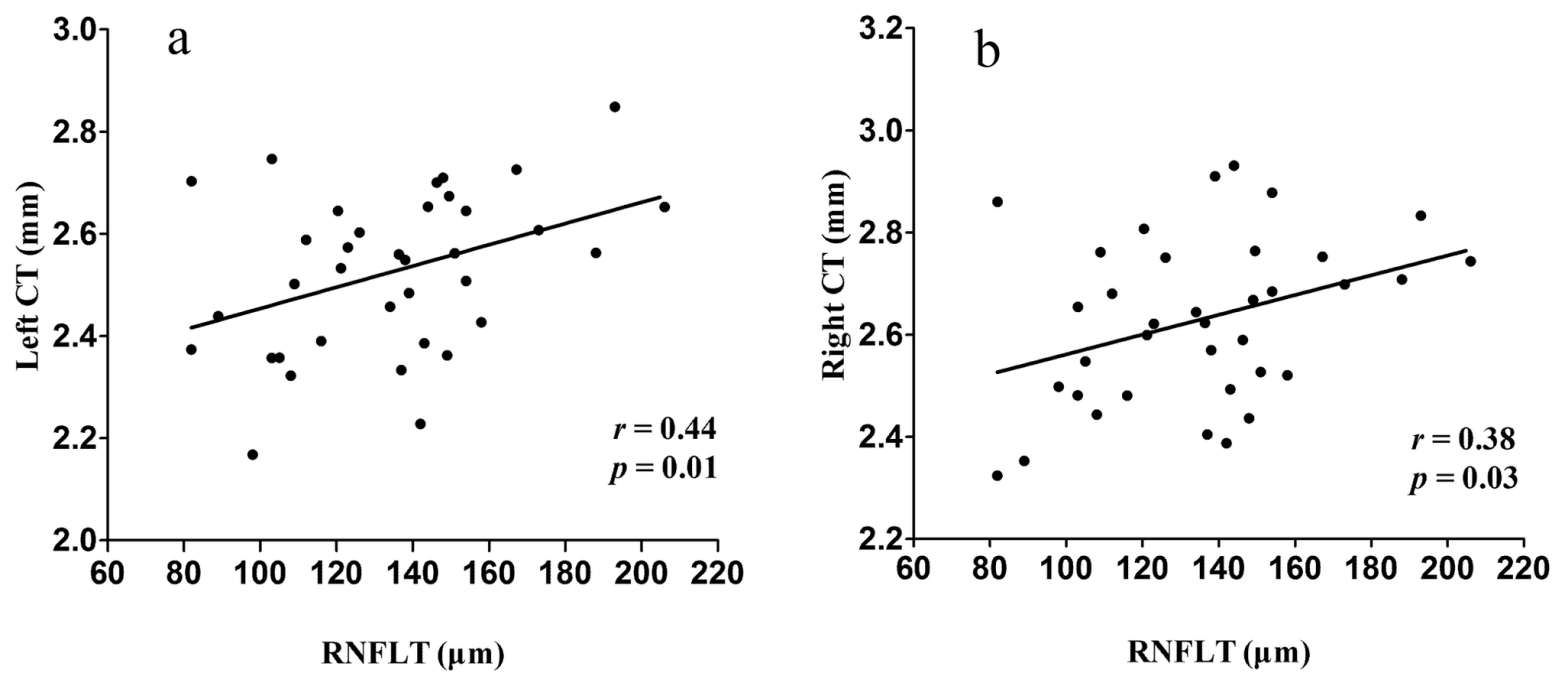
Correlation between cortical thickness (CT) of the calcarine cortex and cumulative retinal nerve fiber layer (RNFL) thickness in POAG patients.

Partial correlation analyses revealed that the left and right cortical thickness around the calcarine sulcus correlated positively with the cumulative RNFL thickness (left, r = 0.44, *p* = 0.01; right, r = 0.38, *p* = 0.03, Figure 2).

### Differences in RNFL Thickness and Cortical Thickness between Mild and Severe Groups

**Figure 3:**
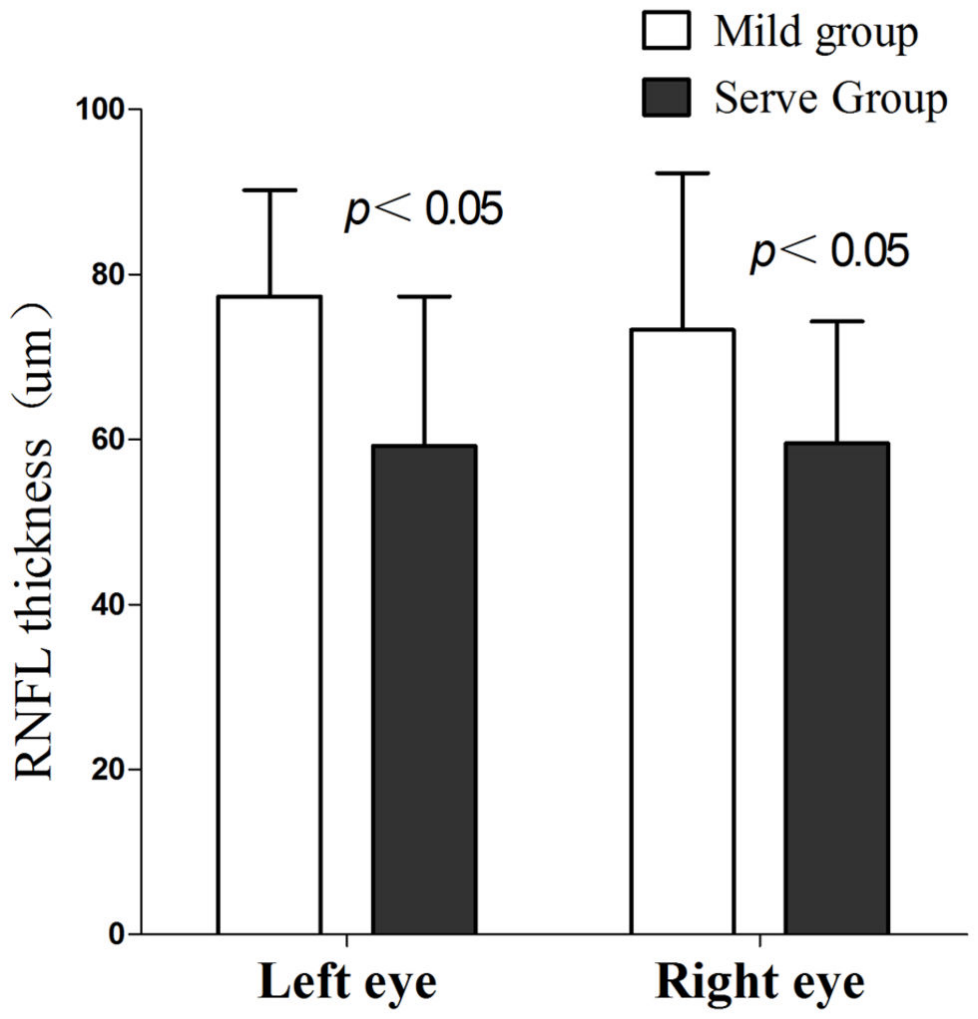
Retinal nerve fiber layer thickness (RNFL) thickness differences in bilateral eyes between mild and severe groups.

**Figure 4:**
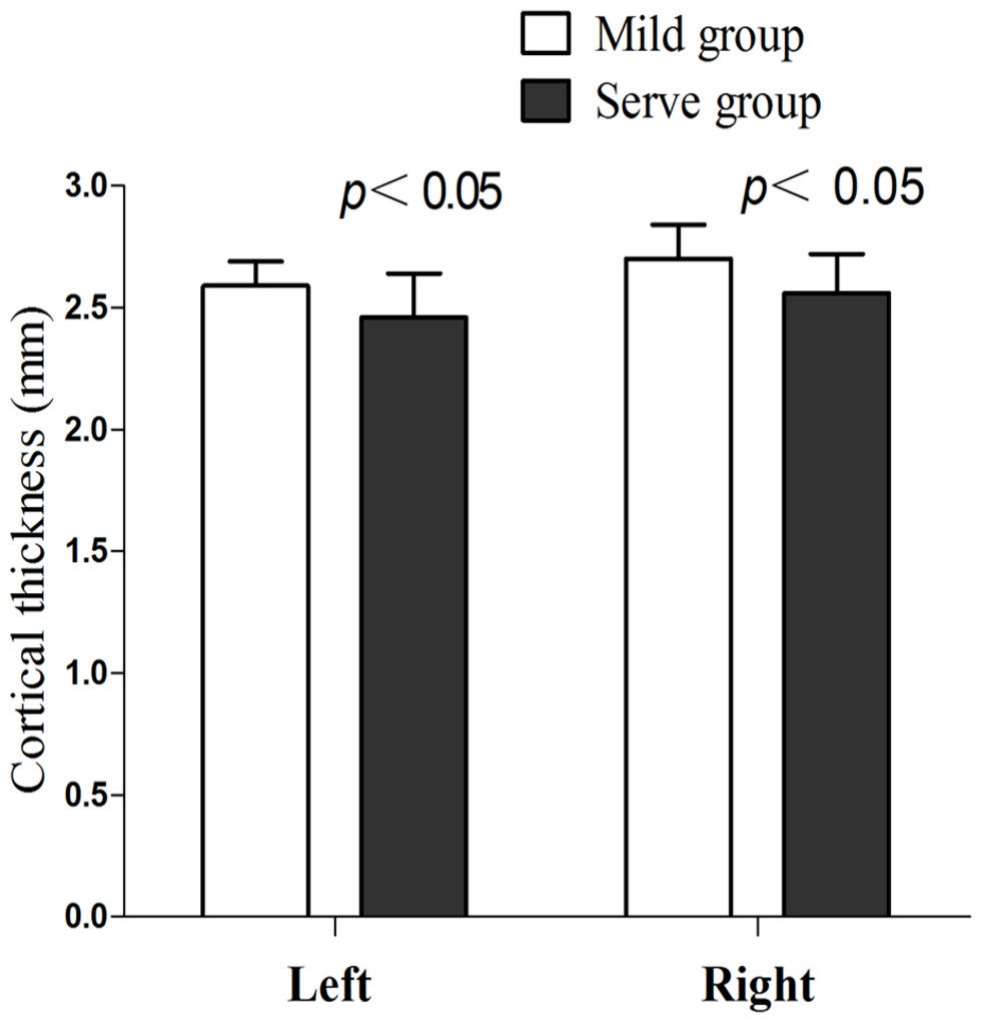
Cortical thickness differences in bilateral calcarine cortex between mild and severe groups.

There was no significant difference in age-, gender-, and education- between mild and severe groups (p > 0.05). The difference in RNFL thickness between the mild and severe groups was significant. Mild vs. severe group: left: 77.3 ± 13.0 versus 59.2 ± 18.1 μm(*p* = 0.001); right: 73.3 ± 19.0 versus 59.2 ± 14.5 μm (*p* = 0.020, Figure 3). In addition, the difference in cortical thickness between the mild and severe groups was also significant in the left and right calcarine cortex. Mild vs. severe group: left: 2.59 ± 0.10 versus 2.46 ± 0.18 mm(*p* = 0.014); right: 2.70 ± 0.14 versus 2.56 ± 0.16 mm (*p* = 0.009, Figure 4).

## Discussion

The present study examined possible changes in cortical thickness and their correlation to RNFL thickness in POAG patients. The results showed that cortical thinning was prominent in the visual-related cortex of POAG patients relative to the control group. In addition, for the first time, the relationship between the thickness of visual cortex and RNFL thickness was also investigated. The results imply that cortical thickness, similarly to RNFL thickness, may reflect underlying neurodegeneration and may be used as an indicator of the severity of glaucoma.

According to our analyses of cortical thickness, patients with POAG showed significant reductions in bilateral visual cortex around the calcarine sulci (left BA 17 and BA 18, right BA17). BA 17 processes visual information from the LGN and projects it to BA18, from which the visual information is projected to the distal ventral or dorsal visual pathway. These results may explain why afferent projections to the visual cortex are largely atrophied in POAG. Consistent with our study, a reduction in visual cortex has also been demonstrated by several VBM studies [9–11]. However, the VBM technique merges information about morphology, size, and position [29], and the ﬁnal measurements include a combination of thickness and cortical folding [30,31] and are therefore less speciﬁc. In contrast, cortical thickness provides a more direct index of cortical morphology [17,18]. This method differentiates between cortices of opposing sulcal walls within the same sulcal bed, thereby enabling more precise measurements in deep sulci [26]. In this study, more pronounced atrophy was observed on the left side (left BA 17 and BA 18, right BA17 only). Using VBM analysis in patients with POAG, Zikou et al. [9] also reported lateralization to the left side but did not report GM changes in the right visual cortex. The lateralization observed in the present study may have resulted from the small sample size.

In addition, the bilateral changes in the visual cortex were located on the anterior half of the calcarine fissures rather than the occipital pole. When visual field defects occur, the corresponding component of the visual cortex is no longer stimulated. The absence of stimulation may cause changes in cortical structure [32,33]. In glaucoma, visual field deterioration typically starts peripherally and progresses towards the fovea at the end stage. The occipital pole area corresponds to the central retina [34]. In this study, the decrease in cortical thickness in POAG patients excluded the occipital pole, possibly because of the preserved central vision of the enrolled patients (patients in stage 5 were not enrolled). These results demonstrate good correspondence between the location of the visual field defect and the location of reduction in cortical thickness. Consistent with this result, previous VBM studies have shown reduced GM volume [11] or density [16] on the anterior half of the medial occipital cortex in glaucoma patients.

The thickness of calcarine cortex correlated positively with the cumulative RNFL thickness, which indicates that cortical thickness of the visual cortex in POAG patients varies consistently with RNFL damage. Several diffusion tensor imaging studies have shown that diffusion parameters of optic nerves [35], optic tracts and optic radiations [28] correlate significantly with RNFL thickness in glaucoma. However, for the visual cortex, the correlation between cortical thickness and RNFL thickness has seldom been studied in human POAG in vivo. These results provide new information on the visual damage and additionally support the use of cortical thickness analysis for studying patients with POAG. Thus, these results imply that analysis of cortical thickness may not only detect abnormalities in the visual cortex in patients with glaucoma but also serve as a biomarker of disease severity.

Our results showed a significant difference in RNFL thickness between mild and severe groups. Based on the correlation between the thickness of the calcarine cortex and the RNFL, we hypothesized that the thickness of the visual cortex may specifically differ between mild and severe groups. The results agree with the cortical thickness findings and imply that the thickness of the visual cortex decreased with increasing severity of POAG. In addition, this difference suggests that the analysis of cortical thickness offers a method as sensitive as the measurement of RNFL thickness to detect POAG damage that may be useful for the earlier diagnosis of POAG in the future.

In glaucoma, the decreased thickness of the visual cortex may as a result of retinal ganglion cell loss. The metabolic activity of astrocytes decreases [7,36] and microglial activation occurs [37] in the visual cortex in parallel with the loss of retinal ganglion cells. And thus, atrophy of the damaged parts of the retina likely propagates by means of transneuronal degeneration via the optic nerve towards the visual cortex. In experimental animal models, this sequence of events has been shown to occur following damage to the retina by elevated intra-ocular pressure [7]. Furthermore, a previous postmortem case report demonstrated reductions in the thickness of the visual cortex in POAG [6].

In addition to alterations in the visual cortex, a significant reduction in cortical thickness was observed in the left middle temporal gyrus (BA37) and fusiform gyrus (BA19), which belong to the distal ventral or dorsal visual pathway, respectively. Our results indicate that the two distal visual pathways were both affected. Previous studies have revealed degeneration of the magnocellular layers (projected to dorsal pathway) and parvocellular layers (projected to ventral pathway) of the LGN [5–7]. However, these studies were based on experimental animal models or postmortem histological examination.

There are some limitations to our study. First, the sample size was relatively small, which reduces both statistical power and the ability to control for confounding variables. Second, LGN damage induced by glaucoma may also result from changes in the visual cortex; thus, the entire visual pathway should be examined using multi-modal MRI in future experiments. Third, as with many previous structural MRI studies, our study used a cross-sectional design that does not permit conclusions on causality. Future longitudinal studies will determine whether cortical thickness may be useful in observing the progression of glaucoma and monitoring the response to treatment.

## Conclusion

In this study, an analysis of cortical thickness revealed cortical thinning in the visual-related cortex in POAG patients, and the thickness of the calcarine cortex was correlated positively with RNFL thickness. Furthermore, the thickness of the RNFL and the bilateral calcarine cortex was significantly different between mild and severe groups. Our findings indicate that cortical thickness analysis may be a sensitive measure for the detection of cortical alterations of the visual system in POAG, which may have great potential for clinical application.
